# Improving therapeutic outcomes in heart failure with reduced nonvalvular ejection fraction: A clinical study of heart failure education intervention

**DOI:** 10.1002/clc.24265

**Published:** 2024-04-29

**Authors:** Xueli Tian, Xiaozeng Li, Qingqing Zhang, Xiangling Qiao, Xin Li, Zijian Zhang

**Affiliations:** ^1^ Department of Pediatrics The Second Affiliated Hospital of Xingtai Medical College Xingtai China; ^2^ Ward One, Department of Cardiology Xingtai Central Hospital Xingtai Hebei China; ^3^ CCU, Department of Cardiology Xingtai Central Hospital Xingtai Hebei China; ^4^ Department of Emergency Xingtai Central Hospital Xingtai Hebei China

**Keywords:** heart failure, heart failure education, reduced nonvalvular ejection fraction, therapeutic effect

## Abstract

**Objective:**

The current study delves into the impact of heart failure education intervention on improving therapeutic outcomes for heart failure (HF) patients with reduced nonvalvular ejection fraction.

**Methods:**

There involved a total of 60 HF patients with non‐valvular ejection fraction reduction who met the inclusion requirements. Patients enrolled were randomly distributed into an observation group and a control group. The observation group received heart failure education intervention, while the control group received conventional intervention. The therapeutic effect, changes in physical indicators, cardiac function indicators, coagulation function, self‐management scale scores, and the incidence of adverse cardiovascular events were meticulously evaluated.

**Results:**

The total effective proportion in the observation group was 96.67%, which was significantly higher than the control group's proportion of 76.67% (*p* < .05). After treatment, several parameters in the observation group showed significant improvements compared to the control group: hs‐CRP, IL‐6, LVEDV value, LVESV value, PT value, APTT value, and TT value were all evidently lower in the observation group. Additionally, the cardiac index, LVEF value, and heart failure self‐management scale fraction were significantly higher in the observation group compared to the control group (*p* < .05). Furthermore, the incidence of adverse cardiovascular events in the observation group was only 6.67%, which was significantly lower than the control group's incidence of 20.00% (*p* < .05).

**Conclusion:**

Heart failure education intervention demonstrates effectiveness in improving the therapeutic outcomes for HF patients and reduced nonvalvular ejection fraction. Additionally, it enhances patients' self‐management abilities. Given these positive results, it is highly recommended to promote and implement HF education intervention in clinical practice.

## Graphical abstract

Heart failure patients with nonvalvular ejection fraction reduction were randomly assigned to either the educational intervention or control intervention group. Patients in educational intervention group exhibited enhancements in effective rate, physical signs, cardiac function, coagulation function, and self‐management scale fraction, as well as a decrease in adverse cardiovascular events.

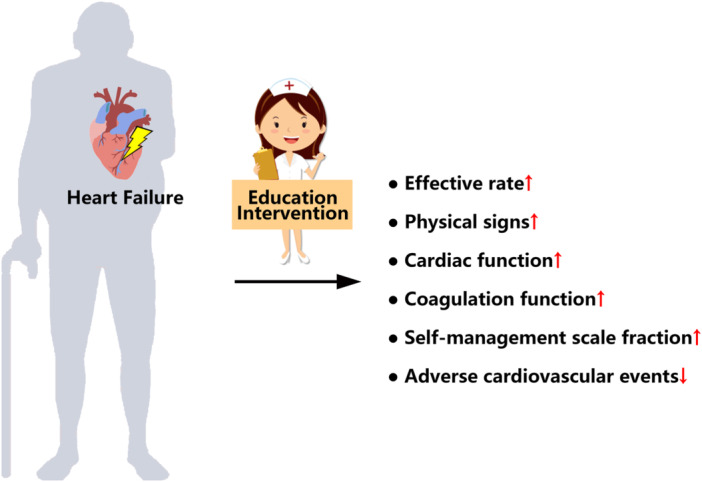

**Abbreviations**
ANOVA: analysis of variance
HF: heart failure
LVEF: left ventricular ejection fraction
LVESV: left ventricular end‐systolic volume

## 1 INTRODUCTION

Heart failure (HF) is a complex syndrome that affects a significant portion of the adult population. This condition is a common cause of reduced quality of life, increased utilization of healthcare resources, and premature mortality.^1^, ^2^ Despite advancements in medical technology and healthcare, the proportion of individuals with cardiovascular diseases surviving has significantly improved, but many eventually progress to HF, leading to a rising incidence of this condition.^3^ Presently, HF represents a critical global public health concern, significantly impacting the quality of life for many patients. Decompensated HF often results in frequent hospitalizations, consuming substantial medical resources and imposing a heavy economic burden on patients. Therefore, the development of sensitive and accurate diagnostic and prognostic evaluation techniques for HF is essential. In the management of HF patients, nurses play an irreplaceable role. Their primary focus is to alleviate clinical symptoms and reduce the frequency of readmissions.^4^ For individuals with HF and reduced nonvalvular ejection fraction, HF education intervention proves to be highly beneficial. Under scientific guidance the theory, this intervention can promote rapid patient recovery and enhancethe patients' understanding of chronic HF and treatment compliance. In light of this, the current study explores the impact of HF education intervention on improving the treatment outcomes in this specific patient group.

## 2 PATIENTS AND METHODS

### 2.1 Patients

The study involved 60 patients with HF and reduced nonvalvular ejection fraction who were treated at our hospital from August 2021 to May 2023. The cohort consisted of 38 males and 22 females, with an average age of (65.13 ± 4.57) years. Inclusion criteria: participants had been diagnosed with chronic HF with in the past 12 months, and their left ventricular ejection fraction (LVEF) was ≤40%. Exclusion criteria: patients with a history of stroke or peripheral arterial embolism, complications of liver, kidney, or coagulation dysfunction, thoseunable to communicate normally, and participants whose inclusion in the study was discontinued for various reasons.

### 2.2 Research group

The 60 eligible participants were randomly allocated into an observation group (*n* = 30) and a control group (*n* = 30). Both groups received conventional drug treatment. Additionally, the observation group received HF education intervention along side the conventional drug treatment, while the control group received conventional intervention without the specific education component.

### 2.3 Conventional drug treatment

According to the 2014 Chinese Guidelines for the Diagnosis and Treatment of HF, medication therapy aimedto ameliorate HF symptoms encompasses strategies such as cardiac strengthening, diuresis (intravenous injection of furosemide 40 mg/d), and vasodilation. Additionally, the gradual introduction of angiotensin‐converting enzyme inhibitors (ACEIs)/angiotensin II receptor blockers (ARBs), β‐receptor blockers, spironolactones, and sodium‐dependent glucose transporters 2 (SGLT‐2) inhibitors is recommended to enhace patient prognosis. In cases where deemed necessary, Western medicines such as Toraxemib tablets (Terasumin) and digitalis tablets are prescribed, with continuous monitoring of cardiac function and potential medication side effects, allowing for adjustments in dosage as needed.

### 2.4 HF education intervention

(1) The HF education intervention involved the establishment of an HF education group, with the head nurse leading the coordination of tasks among team members. This group developed intervention plans and health education manuals tailored to the specific needs of patients and their families. Personalized and targeted interventions were provided to promote gradual changes and improvements in patients' abilities. The HF education content emphasized the following key points: ① Maintaining emotional stability: Encouraging patients to avoid anxiety, depression, tension, and overexcitement to prevent the induction of HF. ② Dietary guidance: Informing patients to adopt a low‐salt, low‐fat diet, consume easily digestible food, opt for smaller, more frequent meals, and avoid full meals. ③ Activity recommendations based on heart function: Tailoring activity levels according to heart function grades—patients with Grade I heart function may not need to restrict daily activities but should avoid excessive physical labor; Grade II patients should not restrict daily activities but increase rest; Grade III patients should limit daily activities and mainly rest in bed, while Grade IV patients should rest in bed and gradually increase activity after improvement. Specific recommendations for elderly patients with congestive HF were provided, emphasizing activity based on the absence of palpitations and shortness of breath, sufficient night time sleep, and the habit of daytime napping. ④ Medication guidelines: Sodium nitroprusside, known for its potent and rapid vasodilatory effects, may induce side effects such asnausea, unease, headache, and hypotension. It is crucial to strictly control the infusion speed during its administration. Patients and their families should be informed not to make any adjustments to the infusion speed independently. When taking digitalis preparations, it is imperative not to arbitrarily adjust the dosage. If the pulse drops below 60 beats/min, patients should cease the medication immediately and seek medical advice. Toxic reaction ssuch as nausea, vomiting, loss of appetite, and changes in vision color (yellow or green) should bed etected promptly and treated without delay. For individuals using diuretics, incorporating potassium‐rich foods like dates, oranges, and bananas into their diet is recommended. Additionally, any symptoms indicating low potassium levels should be reported immediately for timely assessment and management.

(2) In addition to medication guidance, patients were provided witha health education manual tailored to their condition. Detailed one‐on‐one explanations were given based on the manual's contents, covering information on the causes of HF, post‐illness mptoms, and risk factors. Unhealthy behavior habits were analyzed based on patients' daily living habits and self‐management abilities, with relevant opinions and suggestions provided for personalized and targeted intervention measures for HF. The management of HF encompasses various approaches, including general care, pharmacological intervention, surgical procedures, and so forth. It is crucial for patients to seek medical advice for proper guidance on selecting appropriate treatment modalities, thereby preventing the exacerbation of their condition. ① General treatment: Patients with HF should prioritize adequate rest while engaging in moderate activities, avoiding strenuous exercises and overexertion. Maintaining a positive attitude in daily life is essential, steering clear of negative emotions such as anxiety and tenenhance myocardial contractility and improve heart pumping function. Diuretics such as furosemide tablets and hydrochlorothiazide tablets can be administered to reduce cardiac load and alleviate patient discomfort. Beta blockers, including metoprolol succinate sustained‐release tablets and propranolol hydrochloride tablets, may be recommended to slow down heart rate and decrease heart muscle contractility. Additionally, anticoagulant drugs like aspirin enteric‐coated tablets and clopidogrel bisulfate tablets may be advised to prevent thrombosis. ③ Surgical treatment: If HF progresses rapidly and does not respond well to drug therapy, timely hospital intervention is necessary. Surgical options, such as heart transplant surgery or pacemaker implantation may be recommended by healthcare professionals to enhance the patient's quality of life. ④ Other treatments: Complementary measures include oxygen therapy to alleviate HF symptoms. Patients should regulate water intake to avoid increasing cardiac load and can engage in beneficial exercises such as walking and tai chi, as advised by their healthcare provider, to improve overall body resistance.

(3) Regular interviews were conducted with patients and their families to assess their understanding of HF. Daily dietary guidance emphasized the importance of maintaining a diet low in salt, fat, and cholesterol, complemented by suitable protein and vitamin supplementation. Patients were advised to increase their intake of fruits and vegetables, adopting a strategy of consuming smaller, more frequentmeals. The significance of rest and suitable physical exercise in daily life was emphasized, with family member encouraged to accompany patients during their exercise routines. Patients were instructed to cease exercise immediately if they experienced any discomfort in their hearts. (4) With the support of the intervention team, patients and their family members were motivated to join a WeChat group alongside medical stafe for continuous communication. This facilitated timely discussions and addressed any concerns or queries that may arise.^5^, ^6^ The intervention or interview occurred weekly, and communication via WeChat was conducted daily, representing a mobile‐terminal‐type platform.

### 2.5 Observation indicators

#### 2.5.1 Therapeutic effects

The therapeutic effects in both groups were categorized as follows: (1) Marked effect: Patients showed a normalization of LVEF, hs‐CRP, serum IL‐6, cardiac index, and 24‐h urine volume. Their cardiac abilityimproved to Grade 2 or above, and disease symptoms disappeared. (2) Improvement: Patients demonstrated a 50% or greater improvement in the above indicators, and their heart function was restored to Grade 1. (3) Ineffectiveness: Patients showed an improvement rate of less than 50% in the above indicators.

The effective proportion was calculated as follows: Effective proportion = (marked effect + improvement)/total number of patients × 100%.^7^

#### 2.5.2 Changes of signs and indicators

Signs and indicators included LVEF, hs‐CRP, serum IL‐6, and cardiac index. Venous blood was taken from patients in the morning on an empty stomach, and the supernatant was obtained through centrifugation. The levels of hs‐CRP and serum IL‐6 with were determined using a kit.

#### 2.5.3 Cardiac function indexes

Changes in cardiac function indexes between the two groups were compared before and after intervention. All patients underwent electrocardiogram examination, and the examination indexes included LVEF, left ventricular end‐diastolic volume (LVEDV), and left ventricular end‐systolic volume (LVESV).

#### 2.5.4 Coagulation function

A 5 mL of blood sample was taken and centrifuged to obtain the supernatant. PT, APTT, and TT were measured using an automatic coagulation analyzer.

#### 2.5.5 Self‐management scale scores of HF

The HF Self‐Management Inventory (CSMHFI) was devised by Shi Xiaoqing and colleagues at the School of Nursing, Shanghai Jiao Tong University, in 2012. This inventory serves as a tool to measure the health behavior of HF patients in their self‐management endeavors. The self‐management capacity of patients with HF was evaluated using the self‐management capacity scale before intervention, at 3 months post‐intervention, and at 6 months post‐intervention. The scale included 4 dimensions with 20 items, namely, health knowledge, self‐management concept, self‐management responsibility, and self‐management skills. The scoring system consisted of 5 levels, where higher scores indicated better self‐management abilities. The Cronbach's α coefficient of the scale was 0.914, and the reliability was 0.973.^8^

#### 2.5.6 Incidence of adverse cardiovascular events

Adverse cardiovascular events included cardiac death, ventricular tachycardia, recurrent HF, and severe arrhythmia. The incidence proportion was calculated based on the number of occurrences in relation to the total amount of patients.

### 2.6 Statistical analysis

All data in this study were analyzed using SPSS software. Continuous variables were expressed as the mean ± standard deviation. One‐way analysis of variance (ANOVA) with postevent analysis was employed. The Kruskal‐Wallis test was utilized for data with normal distribution, while independent t‐test and Mann‐Whitney U test were employed for further analysis of abnormal distribution data. The χ^2^ test was applied for categorical variables. A significant level of *p* < .05 was considered statistically significant.

## 3 RESULTS

### 3.1 Comparison of therapeutic effects between the two groups

The total effective proportion in the observation group and the control group was 96.67% and 76.67%, respectively (*p* < .05). (Table 1).

**Table 1:** Comparison of therapeutic effects between two groups [*n*(%)].

Group	Number of patients	Significantly effective	Effective	Ineffective	Total effective rate (%)
Observation group	30	19	10	one	96.67
Control group	30	11	12	seven	76.67
χ^2^ value					20.976
*P value*					.001

### 3.2 Comparison of changes in physical signs between the two groups

Before treatment, there were no statistical differences between the two groups (*p* > .05). However, after treatment, the changes in physical signs in the observation group were more favorable than in the control group. Specifically, levels ofhs‐CRP and IL‐6 were significantly lower in the observation group than in the control group, while the cardiac index was significantly higher in the observation group (*p* < .05). (Table 2).

**Table 2:** Comparison of the changes of physical signs between the two groups ($\bar{x}$ ± s).

Group	Treatment time	hs‐CRP (mg/L)	IL‐6 (pg/mL)	Cardiac index [l/(min m2)]
Observation group	Before intervention	15.65 ± 1.19	129.088 ± 25.66	2.11 ± 0.39
	After intervention	5.35 ± 0.98	67.87 ± 5.54	2.87 ± 0.65
	*T* value	12.257	15.548	16.609
	*p value*	.001	.001	.001
Control group	Before intervention	15.87 ± 3.43	130.42 ± 21.98	2.19 ± 0.43
	After intervention	11.08 ± 2.41	102.56 ± 24.54	2.41 ± 0.29
	*T* value	7.879	6.708	6.786
	*p value*	.017	.029	.017

### 3.3 Comparison of cardiac function indexes between the two groups

Before the intervention, there were no significant differences in LVEF, LVEDV, and LVSV between the observation group and the control group (*p* > .05). After the intervention, LVEF values increased, and the LVEF values of patients in the observation group were significantly higher than in the control group (*p* < .05). However, LVEDV values and LVESV values decreased, and the values in the observation group were significantly lower than in the control group (*p* < .05). (Table 3).

**Table 3:** Comparison of cardiac function indexes between the two groups ($\bar{x}$ ± s).

Index	Point of time	Observation group (*n* = 30)	Control group (*n* = 30)	*T* value	*p value*
LVEF (%)	Before intervention	39.98 ± 2.34	39.33 ± 1.02	0.754	.551
	After intervention	67.57 ± 3.42	50.56 ± 1.45	16.790	.001
LVEDV (mm)	Before intervention	61.19 ± 3.87	61.64 ± 3.33	0.483	.149
	After intervention	37.89 ± 4.01	45.89 ± 2.32	17.324	.001
LVESV (mm)	Before intervention	54.89 ± 5.62	54.92 ± 4.90	0.656	.331
	After intervention	29.14 ± 3.25	43.45 ± 2.08	11.869	.001

### 3.4 Comparison of coagulation function between two groups

Before the intervention, there were no significant differences in PT, APTT, and TT between the observation group and the control group (*p* > .05). After the intervention, the values of PT, APTT, and TT decreased in both groups, with significantly lower values observed in the observation group compared to the control group (*p* < .05). (Table 4).

**Table 4:** Comparison of coagulation function between the two groups ($\bar{x}$ ± s).

Index	Time	Observation group (*n* = 30)	Control group (*n* = 30)	*T* value	*p value*
PT(s)	Before intervention	25.20 ± 2.56	24.53 ± 0.12	0.896	.276
	After intervention	12.23 ± 1.43	16.16 ± 1.02	15.489	.001
APTT(s)	Before intervention	27.86 ± 3.42	26.99 ± 3.41	0.337	.590
	After intervention	12.16 ± 1.02	21.65 ± 3.02	15.362	.001
TT(s)	Before intervention	23.43 ± 2.98	24.57 ± 1.58	0.909	.443
	After intervention	15.22 ± 2.01	18.92 ± 2.03	18.451	.001

### 3.5 Comparison of self‐management scale scores of HF between the two groups

Before the intervention, there were no significant differences in the scores of health knowledge, self‐management concept, self‐management responsibility, and self‐management skills between the observation group and the control group (*p* > .05). However, after the intervention, the self‐management scale scores in both groups improved, with significantly higher scores observed in the observation group compared to the control group (*p* < .05). (Table 5).

**Table 5:** Comparison of self‐management scale scores of heart failure between the two groups ($\bar{x}$ ± s).

Index	Point of time	Observation group (*n* = 30)	Control group (*n* = 30)	*T* value	*p value*
Health knowledge	Before intervention	19.08 ± 3.43	18.54 ± 2.16	0.129	.342
	After intervention	63.72 ± 5.09	47.09 ± 3.79	10.923	.001
Self‐management concept	Before intervention	23.26 ± 2.90	26.64 ± 4.32	0.509	.225
	After intervention	50.64 ± 2.09	43.21 ± 2.36	15.568	.001
Self‐management responsibility	Before intervention	10.98 ± 1.66	12.18 ± 1.66	0.407	.358
	After intervention	44.38 ± 4.58	29.56 ± 4.58	10.327	.001
Self‐management skills	Before intervention	30.67 ± 3.10	35.33 ± 3.09	0.614	.378
	After intervention	74.38 ± 3.14	54.31 ± 4.45	12.312	.001

### 3.6 Comparative analysis of the incidence of adverse cardiovascular events between the two groups

The incidence of adverse cardiovascular events in the observation group and the control group was 6.67% and 20.00%, respectively. Notably, the observation group had a significantly lower incidence compared to the control group (*p* < .05). (Table 6).

**Table 6:** Comparative analysis of the incidence of adverse cardiovascular events between the two groups [*n* (%)].

Group	Cardiogenic death	Ventricular tachycardia	Heart failure recurrence	Severe arrhythmia	Total incidence (%)
Observation group (*n* = 30)	one	one	0	0	2 (6.67)
Control group (*n* = 30)	2	one	one	2	6 (20.00)
χ2 value					0.768
*p value*					.554

## 4 DISCUSSION

HF is a significant health challenge, affecting over 5 million Americans, and is particularly prevalent in elderly patients. Its onset is attributed to a decline in myocardial contractility, leading to inadequate cardiac output to meet the body's metabolic demands, resulting in adverse symptoms such as pulmonary circulation disturbances.^9^, ^10^ In cases of HF with reduced nonvalvular ejection fraction, timely and effective treatment is essential, as failure to control the condition promptly may lead to complications that severely jeopardize the patients' life.^11^ While medications for treating HF with reduced nonvalvular ejection fraction can provide temporary relief of clinical symptoms in patients, their long‐term treatment outcomes are not always optimal, impacting patients' prognosis and overall quality of life.^12^, ^13^, ^14^

Following HF education intervention, the treatment outcomes for HF patients showed significant improvement, aligning with findings from previous studies.^15^, ^16^ This study found that the total effective rate of patients who received HF education intervention was higher than those who did not receive such intervention. Siennicka et al. conducted a study on the long‐term effects of HF education intervention instable HF patients and observed significant improvements in their quality of life and functional motor abilities.^17^ The researchers conclude that HF education intervention is an effective approach to enhancing both the quality of life and treatment outcomes. Similarly, Kosiborod et al. reported that the an 8‐week education intervention for HF patients resulted in significant improvements in nonvalvular ejection fraction and self‐management levels.^18^ Furthermore, it is evident that factors such as exercise training can contribute to improving therapeutic outcomes by addressing edema and other discomforting symptoms in HF patients.^19^, ^20^ Additionally, paying attention to diet may also play a role in enhancing treatment effectiveness.^21^ Moreover, providing consultation through instant messaging services allows for early symptom monitoring, prompt identification, and immediate emotional support, thereby potentially improving treatment outcomes.^22^

Our research depicted significant improvements in LVEF, LVEDV, and LVSV as heart function indexes in HF patients after the intervention. These results are consistent with previous studies. Myocardial cell transformation and enlargement of the heart cavity are key factors in HF progression. Clinical trials have shown that LVEF is an independent risk factor for predicting HF prognosis when the heart cavity is enlarged.^23^, ^24^ Leclercq et al. conducted a comparative study between HF education intervention and standard outpatient‐based intervention in HF patients with reduced nonvalvular ejection fraction, and both intervention approaches significantly improved the treatment outcomes.^25^ However, patients in the HF education intervention exhibited better compliance and significantly improved self‐management abilities compared to the standard intervention group. Further more, our study revealed that after the HF intervention, patients demonstrated higher scores in health knowledge, self‐management concept, self‐management responsibility, and self‐management skills compared to the control group.

In terms of coagulation ability, plasma prothrombin time primarily reflects the ability of the exogenous coagulation pathway. APPT is the main index for assessing endogenous coagulation factor deficiency and is common in patients with hypopfibrinogen and abnormal fibrinogen levels. Our research demonstrated significantly reduced levels of PT, APTT, and TT in HF patients with reduced nonvalvular ejection fraction after intervention. As a result, HF intervention effectively improved the coagulation indexes, further enhancing the clinical prognosis of patients and promoting therapeutic effectiveness.

Taken together, HF education intervention is a valuable approach to enhance the therapeutic outcomes for HF patients with reduced nonvalvular ejection fraction. Moreover, it improves the self‐management abilities of patients, making it a worth while strategy for clinical application.

## AUTHOR CONTRIBUTIONS

Xueli Tian contributed to the conception and design of the study. Xiaozeng Li performed the experiments, collected and analyzed data. Xin Li wrote the manuscript. Xueli Tian and Xiaozeng Li revised the manuscript. All authors reviewed and approved the final version of the manuscript.

## CONFLICT OF INTEREST STATEMENT

The authors declare that they have no competing interests.

## Data Availability

The datasets analyzed during the current study are not publicly available due to the personal privacy but are available from the corresponding author on reasonable request.
